# Clinical applicability of functional hemodynamic monitoring

**DOI:** 10.1186/2110-5820-1-35

**Published:** 2011-08-25

**Authors:** Xaime García, Michael R Pinsky

**Affiliations:** 1 Department of Critical Care Medicine, University of Pittsburgh, Pittsburgh PA 15261, USA

## Abstract

Recent interest in functional hemodynamic monitoring for the bedside assessment of cardiovascular insufficiency has heightened. Functional hemodynamic monitoring is the assessment of the dynamic interactions of hemodynamic variables in response to a defined perturbation. Accordingly, fluid responsiveness can be predicted in a quantities fashion by measuring as arterial pulse pressure variation and left ventricular stroke volume variation during positive pressure breathing or the change in cardiac output response to a passive leg raising maneuver. However, volume responsiveness, though important, reflects only part of the overall spectrum of functional physiological variables that can be measured to define physiologic state and monitor response to therapy. Dynamic tissue O_2 _saturation (StO_2_) responses to complete stop flow conditions, which can be created by measuring hand StO_2 _and occluding flow with a blood pressure cuff, assesses cardiovascular sufficiency and microcirculatory blood flow distribution. Furthermore, these measures can be made increasingly more sensitive and specific if coupled to other "traditional" measures of organ perfusion, such as blood lactate levels.

## Introduction

Recently, increased interest in a more proactive use of monitoring technologies has emerged, using the response to the measured variables to a defined stress to unveil the physiological state of the subject. This entire field when applied to the assessment of cardiovascular state is referred to as functional hemodynamic monitoring. Within this context, we can define functional hemodynamic monitoring as the assessment of the dynamic interactions of hemodynamic variables in response to a defined perturbation [1]. Such dynamic responses result in emergent parameters of these commonly reported variables that greatly increase the ability of these measures to define cardiovascular state and predict response to therapy. At the present time, the primary types of functional hemodynamic monitoring for which clinical trials have shown clinical usefulness are related to predicting volume responsiveness and identifying occult cardiovascular insufficiency (compensated shock). The primary form of this approach, predicting volume responsiveness, was reviewed recently in this journal, so will only be briefly described in this article. However, like any form of monitoring parameter, its sensitivity and specificity improves if its pretest probability is higher. Thus, a fundamental concept often ignored by proponents of functional hemodynamic monitoring approaches is the integration of other clinical variables, such as the clinical condition, serum lactate levels, etc., a priori into the decision analysis of cardiovascular instability and its response to therapy. In essence, functional hemodynamic monitoring, though profoundly insightful in its values, is only another parameter that needs to be integrated into the greater view of patient care if its use is to realize its full potential.

### Predicting volume responsiveness

A primary resuscitation question is whether the patient will increase their cardiac output in response to intravascular volume infusion. Volume responsiveness has been arbitrarily defined as ≥15% in cardiac output in response to a 500-ml bolus fluid challenge. Although the presence of fluid responsiveness in a subject does not equate for the need to give fluids, it does define that if fluids are infused cardiac output will increase. Many studies have validated, during recent years, the usefulness of some methods that give the clinician the ability to predict whether a patient is going to respond to the volume infusion as a primary step of the hemodynamic resuscitation. Some of the most commonly used methods are those based in changes in left ventricular output during positive pressure ventilation, such pulse pressure variation (PPV) and stroke volume variation (SVV). During the inspiratory phase of positive pressure ventilation, intrathoracic pressure increases passively, increasing right atrial pressure and causing venous return to decrease, decreasing right ventricular output, and after two or three heart beats, left ventricular output if both ventricles are volume responsive [2]. Thus, in preload-dependent patients cyclic changes in left ventricular stroke volume and its coupled arterial pulse pressure are seen and the magnitude of the changes is proportional to volume responsiveness. The associated SVV and PPV are quantified in various ways depending on whether these are measured by minimally invasive cardiac output monitors (e.g., PiCCO, LiDCO, FloTrac) or by direct examination of the pressure or flow profiles. In general both are defined as the ratio of the maximal minus the minimal values to the mean values, usually averaged over 3 or more breaths. Numerous studies have documented that a SVV >10% or a PPV >13-15% on a tidal volume of 8 ml/kg or greater is highly predictive of volume responsiveness [2-4].

Although powerful diagnostic tools, these parameters are highly dependent on the cyclic changes in intrathoracic pressure being regular and great enough to alter central venous pressure. Thus, tidal volumes of ≤6 ml/kg or the imposition of variable spontaneous inspiratory efforts often result in false-negative PPV and SVV values. Moreover, all of these techniques assume a fixed heart rate, so in the setting of atrial fibrillation or frequent premature ventricular contractions, these measures become inaccurate. In these settings, one can always perform a passive leg raising maneuver [5].

Fluid therapy is one of the first steps in the goal-directed therapy in clinical guidelines of hemodynamic resuscitation of patients in shock [6,7]. Thus, an adequate assessment of fluid responsiveness should improve the therapy. A study conducted in high-risk surgery patients showed that volume loading guided to the goal of PPV minimization improves postoperative outcome and decreases length of hospital stay [8]. Patients in the interventional group received more fluid than the control group and had less postoperative complications, lower duration of mechanical ventilation, and shorter stay in the intensive care unit. Similarly, Pearse et al. [9] conducted a study to evaluate the effect of postoperative goal-directed therapy in postoperative high-risk patients. Targeting an oxygen delivery index > 600 mL/kg/min, they were able to reduce both postoperative complications and median duration of hospital stay.

### Identification of cardiovascular insufficiency

Cardiovascular insufficiency is characterized by an inadequate O_2 _delivery relative to the metabolic demands. Shock can be, in the early stages, compensated by autonomic mechanisms, such regional vasoconstriction, in an attempt to maintain central blood pressure and vital organ perfusion above an anaerobic threshold. In this stage of compensated shock, microcirculatory measures, such as arterial pressure or cardiac output often are inside the range of values defined as normal and, therefore, insensitive as early predictors of subsequent decompensation due to the increased risk of tissue ischemia and subsequent development of multiorgan failure and death. However, microcirculation alterations in muscle and skin blood flow already occur during these early stages and measures of tissue cardiovascular reserve should be a sensitive early warning measure of impending cardiovascular collapse. Thus, a valid method to assess the microcirculatory status, such as the noninvasive measurement of tissue oxygen saturation (StO_2_) when coupled with a functional hemodynamic monitoring test, such as the vascular occlusion test (VOT), may allow early identification of compensated circulatory shock and thus guide initial resuscitation efforts.

Noninvasive measurement of StO_2 _using near-infrared spectroscopy has been shown as a valid method to assess the microcirculation status, especially in septic and trauma patients. The absolute StO_2 _value has a limited discriminating capacity because StO_2 _remains within the normal range until shock is quite advanced. But the addition of a dynamic ischemic challenge, such as the VOT, improves and expands the predictive ability of StO_2 _to identify tissue hypoperfusion [10]. The VOT measures the effect of total vascular occlusion-induced tissue ischemia and release on downstream StO_2_. StO_2 _is measured on the thenar eminence and transient rapid vascular occlusion of the arm by sphygmomanometer inflation to 30 mmHg above systolic pressure is performed either for a defined time interval, usually 3 min, or until StO_2 _declines to some threshold minimal value, usually 40%. The deoxygenation rate (DeO_2_) reflects the local metabolic rate and mitochondrial function, and the rate of reoxygenation rate (ReO_2_) reflects local cardiovascular reserve and microcirculatory flow.

There is strong evidence for microcirculatory failure during shock to be a major component of the end-organ dysfunction seen. Such microcirculatory dysfunction can be characterized by oxygen shunting, vasoconstriction, thrombosis, and tissue edema. As a result of these combined microcirculatory events, the flow distribution within the tissue is impaired [11]. It has been shown that these microcirculatory alterations improve rapidly in septic shock survivors, whereas patients dying by organ failure have a lower percentage of perfused small vessels [12].

The hypothesis that the alterations in VOT StO_2 _response are related to the outcome has been proved in patients with severe sepsis or septic shock by Creteur et al. [13]. Furthermore, when comparing hemodynamically stable patients without infection (controls) and healthy volunteers, these differences in the septic patients were striking. Using near-infrared spectroscopy VOT StO_2_, they assessed the slope of increase in StO_2 _release as well as the difference between the maximum StO_2 _and the StO_2 _baseline (Δ). Both the slope of ReO_2 _and the Δ were significantly lower in septic patients than in control subjects and healthy volunteers. In the sample of septic patients, the slopes also were significantly lower in the ones who had cardiovascular insufficiency. ReO_2 _slopes were higher in survivors than in nonsurvivors and also tended to increase during resuscitation in survivors but not in nonsurvivors. Finally, the ReO_2 _slope was found to be a good predictor of ICU death, with a cutoff value of 2.55%/sec (sensitivity 85%, specificity 73%). These data confirm that the alterations in VOT StO_2 _ReO_2 _are related more to the sepsis process itself and its severity than to mean arterial pressure or vasopressor agent's dose. Importantly, the magnitude of this ReO_2 _slope alteration is directly related to the septic disease and their presence in the first 24 hours of septic process and their persistence of delayed ReO_2 _slope is related to the patient's outcome. Still, if the StO_2 _ReO_2 _does reflect inadequate tissue perfusion then it also should be sensitive of an impending cardiovascular insufficiency state (compensated shock) if matched with other static measures of tissue ischemia.

To address this issue, Guyette et al. [14] measured both the VOT StO_2 _as baseline serum lactate, known to define existing cardiovascular insufficiency in trauma, in a cohort of trauma patients during the air transport to the Trauma Center. This study was designed to determine whether the StO_2 _measurement, including a VOT, was feasible in the prehospital environment and useful to predict in-hospital death and intensive care unit (ICU) admission. Not surprisingly, they did not find differences in baseline StO_2 _between survivors, nonsurvivors, and patients admitted to the ICU, and they showed significant differences in DeO_2 _and ReO_2 _slopes between survivors and nonsurvivors, as well as between patients who need ICU admission and patients who did not. Furthermore, only one of the five patient deaths in their sample had prehospital vitals signs that would have met the protocolized criteria for resuscitation (heart rate >120 bpm, systolic blood pressure <90 mmHg). Importantly, serum lactate alone was no better than lowest systolic pressure in predicting those in need of life-saving interventions or death, but if the baseline serum lactate was >1.7 mmol/dl the ReO_2 _was 100% specific for the need of life-saving interventions. This study shows the usefulness of the microcirculation dynamic assessment in the early stages of the trauma injury, when cardiovascular insufficiency is not suspected with the macrocirculatory indexes, providing the possibility to start early the appropriate treatment and decide the in-hospital disposition.

These studies show that microcirculation status, measured by a dynamic test, such the StO_2 _VOT, can be more accurate than the microcirculatory and static classic values assessing the cardiovascular insufficiency in patients with shock. A study conducted by Vallée et al. [15] proved the hypothesis that in septic patients when the goal of the resuscitation bundles (central venous oxygen saturation (SvcO_2_) >70%) has been already achieved, an index of tissue perfusion, such as the venous-to-arterial carbon dioxide difference [P(cv-a)CO_2_], could be useful to identify those patients who still were inadequately resuscitated. Patients with [P(cv-a)CO_2_] higher than 6 mmHg had a lower cardiac index, a larger clearance of lactate, and a slower decrease of SOFA score in the first 24 h than patients with a [P(cv-a)CO_2_] lower than 6 mmHg [15].

The presence of cardiovascular insufficiency in patients with shock also can be assessed by metabolic products, such as lactate or strong ion difference (SID). The early presence of high levels of plasma lactate is associated with higher in-hospital mortality in patients presenting with circulatory shock [16,17]. Moreover, those patients who are able to decrease their lactate levels with the hemodynamic resuscitation procedures have a better outcome than those who are not. Thus, it would be reasonable to target lactate levels as an endpoint of the hemodynamic resuscitation interventions as a way to early know when a patient is being correctly resuscitated. A trial that focused on reaching a SvcO_2 _>70% and lactate concentration ≤2 mmol/L by improving the oxygen delivery in postsurgical patients whose lactate levels were high or did not decrease showed a shorter hospital stay and lower morbidity [18]. A more recent study by Jones et al. [19] focused on comparing two early sepsis resuscitation protocols: one was designed to normalize central venous pressure (CVP), mean arterial pressure (MAP), and lactate clearance of at least 10%, and another with SvcO_2 _as a goal, as well as CVP and MAP, showed no difference for in-hospital mortality.

In addition to the creation of lactate, tissue hypoperfusion due to cardiovascular insufficiency induces formation of anions as results of anaerobic metabolism and, finally, acidosis. The anion gap and, especially, the SID calculated as the difference between fully dissociated anions and cations, may help to evaluate those cases of metabolic disorders with acidosis adding the possibility to quantify the contribution of each metabolic disorder and to know the source of the acidosis. The approach, based on the principles described by Stewart [20], proposes the SID, as one of the three factors (together with the total weak acid difference and the PaCO_2_) that independently determine the pH. It has been proven that the SID is useful to identify patients with major acid-base disturbances even when the standard base excess is normal [21]. Nevertheless, it has not been reflected in superiority against standard base excess predicting mortality, nor tested as goal of directed therapy in shock patients [21,22].

## Conclusions

Functional hemodynamic monitoring is the pluripotential approach to interpolation of physiological data in a proactive form. It has acquired a strong foothold in assessment of volume responsiveness for the management of the critically ill patient, showing a high applicability in shock resuscitation, but needs to be considered within the broader aspects of risk stratification to reach its full potential. Because early goal-directed therapy algorithms need to resuscitate to circulatory sufficiency, defining endpoints become as important as defining treatments to initiate. This field is rapidly expanding and has potential application for assessment of regional perfusion and function and across all acute care disciplines.

## Competing interests

Xiame Garcia declares that he has no competing interests. Michael R. Pinsky is a consultant for Edwards LifeScieince, LiDCO Ltd, Cheetah Medical, Inc., has stock options with LiDCO Ltd., and has been paid to present a lecture by Edwards LifeScieinces, LiDCO Ltd and Hutchinson, Industries.

## Authors' contributions

XG wrote the original version of the manuscript after consultation with MRP. MRP defined the scope of the manuscript and wrote all the revisions of the manuscript to final form.
